# Supplementary material for: Osteoporosis Is Associated with an Increased Risk of Colorectal Neoplasms Regardless of Sex: Nationwide Population-Based Cohort Study
Source: Diagnostics (Basel). 2024 Mar 21;14(6):666. doi: 10.3390/diagnostics14060666 (PMC10968966; doi:10.3390/diagnostics14060666)
Supplement: Supplementary file 1 [file diagnostics-14-00666-s001.zip › diagnostics-2882598-supplementary.pdf]

Supplementary Table S1. NHSP participants among NHIS cohort who underwent BMD (n=35,099)

Variables	Total, n (%)	Osteoporosis, n (%)		p value
	(n=35,099)	No (n=4,609)	Yes (n=30,490)	
Colonic neoplasms				< 0.0001
No	31,001 (88.3)	4,190 (90.9)	26,811 (87.9)	
Yes	4,098 (11.7)	419 (9.1)	3,679 (12.1)	
Low grade adenoma				< 0.0001
No	31,560 (89.9)	4,231 (91.8)	27,329 (89.6)	
Yes	3,539 (10.1)	378 (8.2)	3,161 (10.4)	
High grade adenoma/CIS				0.3368
No	35,015 (99.8)	4,595 (99.7)	30,420 (99.8)	
Yes	84 (0.2)	14 (0.3)	70 (0.2)	
Non-invasive CRN				< 0.0001
No	31,501 (89.7)	4,222 (91.6)	27,279 (89.5)	
Yes	3,598 (10.3)	387 (8.4)	3,211 (10.5)	
Invasive CRC				< 0.0001
No	34,373 (97.9)	4,555 (98.8)	29,818 (97.8)	
Yes	726 (2.1)	54 (1.2)	672 (2.2)	
Sex				< 0.0001
Male	5,524 (15.7)	2,151 (46.7)	3,373 (11.1)	
Female	29,575 (84.3)	2,458 (53.3)	27,117 (88.9)	
Age, years				< 0.0001
50–54	5,017 (14.3)	1,354 (29.4)	3,663 (12.0)	
55–59	5,992 (17.1)	969 (21.0)	5,023 (16.5)	
60–64	6,603 (18.8)	688 (14.9)	5,915 (19.4)	
65–69	7,578 (21.6)	588 (12.8)	6,990 (22.9)	
70–74	5,627 (16.0)	557 (12.1)	5,070 (16.6)	
75–79	2.905 (8.3)	302 (6.5)	2,603 (8.5)	
**≥ 80**	1,377 (3.9)	151 (3.3)	1,226 (4.0)	
CCI				< 0.0001
0	8,523 (24.3)	1,084 (23.5)	7,439 (24.4)	
1	9,349 (26.6)	1,068 (23.2)	8,281 (27.2)	
2	7,318 (20.9)	906 (19.7)	6,412 (21.0)	
3 or more	9,099 (28.2)	1,551 (33.6)	8,358 (27.4)	
Income level				0.2765
0–3	8,739 (24.9)	1,120 (24.3)	7,619 (25.0)	
4–7	11,983 (34.1)	1,552 (33.7)	10,431 (34.2)	
8–10	14,377 (41.0)	1,937 (42.0)	12,440 (40.8)	
BMI group				< 0.0001
≤ 22.9	14,100 (40.2)	1,521 (33.0)	12,579 (41.2)	
23–24.9	8,935 (25.4)	1,230 (26.7)	7,705 (25.3)	
≥ 25	12,064 (34.4)	1,858 (40.3)	10,206 (33.5)	
Smoking				< 0.0001
non	30,808 (87.8)	3,196 (69.3)	27,612 (90.6)	
ex-	2,209 (6.3)	870 (18.9)	1,339 (4.4)	
current	2,082 (5.9)	543 (11.8)	1,539 (5.0)	
Alcohol				< 0.0001
No	28,714 (81.8)	2,905 (63.0)	25,809 (84.6)	
Yes	6,385 (18.2)	1,704 (37.0)	4,681 (15.4)	

BMD, bone mineral density; BMI, body mass index; CCI, Charlson comorbidity index; CIS, carcinoma in situ; CRC, colorectal cancer; CRN, colorectal neoplasm; NHSP, National Health Screening Program; NHIS, National Health Insurance Service
